# Metatranscriptomic characterization of active microbial communities in strawberry hydroponic drainage effluent

**DOI:** 10.3389/fmicb.2026.1909518

**Published:** 2026-08-03

**Authors:** Mi-Ri Park, Miah Bae

**Affiliations:** Region-Specific Industries Fostering Division, Cheorwon Plasma Research Institute, Cheorwon-gun, Gangwon-do, Republic of Korea

**Keywords:** hydroponic drainage effluent, metatranscriptomics, microbial ecology, microbiome, strawberry hydroponics

## Abstract

Hydroponic cultivation systems improve water and nutrient use efficiency; however, little is known about the active microbial communities inhabiting hydroponic drainage effluent. This study employed metatranscriptomic sequencing to characterize active microbial communities present in drainage effluent collected from strawberry cultivation beds within a commercial recirculating hydroponic system. Drainage effluent samples were collected during the spring and winter cultivation periods and subjected to RNA-based metatranscriptomic analysis. Following quality filtering, *de novo* assembly, and taxonomic classification, bacterial, fungal, and viral-associated transcripts were analyzed to characterize active microbial communities within the drainage environment. Metatranscriptomic sequencing generated 65.2 million and 53.2 million paired-end reads from the spring and winter samples, respectively. Taxonomic classification revealed distinct microbial profiles between the two analyzed drainage samples. Bacterial transcripts represented the dominant classified component in both samples. The spring sample exhibited a relatively diverse bacterial community composed of multiple taxa, whereas the winter sample was strongly dominated by *Serratia marcescens* and *Serratia proteamaculans*. Fungal community composition also differed between samples, with a greater representation of yeast-associated fungi in the winter sample. Viral-associated transcripts were detected in both samples and were primarily represented by bacteriophage-related sequences. A large proportion of transcripts remained unclassified, particularly in the spring sample, highlighting the limited representation of hydroponic drainage microorganisms in current reference databases. Although the study was limited to a single commercial production site, the findings should be interpreted as site-specific observations rather than representative characteristics of strawberry hydroponic systems in general. Nevertheless, the study provides an initial metatranscriptomic characterization of active microbial communities inhabiting strawberry hydroponic drainage effluent under commercial cultivation conditions and establishes a baseline dataset for further comparative investigations involving multiple hydroponic production systems. These findings provide baseline information on active microbial and viral assemblages associated with hydroponic drainage effluent and demonstrate the utility of metatranscriptomics for characterizing microbial communities in recirculating cultivation systems.

## 1 Introduction

Strawberry (*Fragaria* × *ananassa* Duch.) is one of the most economically important horticultural crops cultivated in protected greenhouse environments worldwide (Giampieri et al., 2012). In recent years, hydroponic strawberry cultivation systems have been increasingly adopted because they enable efficient nutrient management, stable crop production, and reduced dependence on soil-based cultivation (Kouloumprouka Zacharaki et al., 2024). In particular, recirculating hydroponic systems allow nutrient solutions to be reused, thereby improving water and fertilizer use efficiency while reducing environmental discharge (Tanemura and Ohyama, 2023). As strawberry production continues to intensify in controlled-environment agriculture, understanding the biological processes occurring within recirculating cultivation systems has become increasingly important for sustainable crop management (Lema-Rumińska et al., 2021). Despite these agronomic advantages, the continuous circulation of nutrient solutions may also facilitate the accumulation and persistence of diverse microorganisms within the cultivation system (Dong et al., 2020). Because microbial communities are influenced by cultivation practices, environmental conditions, and hydroponic system design, microbial profiles may vary considerably among different hydroponic production systems.

Hydroponic drainage effluents contain a wide range of biological and chemical components derived from plant root exudates, microbial metabolites, and suspended organic materials (Dyśko et al., 2020). These drainage environments may serve as unique ecological niches that support diverse microbial assemblages, including bacteria, fungi, and viruses (Guevara et al., 2025). In recirculating cultivation systems, microorganisms associated with plant roots and nutrient solutions can continuously interact and disperse throughout the system, potentially influencing microbial succession, biofilm formation, nutrient turnover, and overall system stability (Qu et al., 2021). In addition, environmental conditions commonly associated with greenhouse strawberry cultivation, including temperature, humidity, and nutrient composition, may further shape microbial community composition within hydroponic drainage environments (Schmautz et al., 2021).

Previous studies investigating hydroponic microbial communities have primarily focused on bacterial and fungal populations using culture-dependent methods or DNA-based profiling approaches (Guevara et al., 2025). Although these studies have provided valuable information regarding microbial diversity in hydroponic systems, they do not necessarily reflect metabolically active microbial populations. In contrast, metatranscriptomic analysis enables the investigation of actively transcribed RNA molecules and can therefore provide insights into biologically active microbial communities present in environmental samples (Nakagawa et al., 2023). This approach may be particularly useful for understanding active microbial ecology in hydroponic systems, where environmental conditions and nutrient availability dynamically influence microbial activity. However, active microbial communities inhabiting strawberry hydroponic drainage effluent remain largely unexplored, and little is known about the microorganisms that are actively transcribing genes within these cultivation environments.

In addition to bacteria and fungi, viruses are important components of plant-associated microbial communities in aquatic and hydroponic environments (Trivedi et al., 2020). These microbial groups interact within hydroponic systems and collectively shape microbial community dynamics and ecosystem functioning (Dong et al., 2020; Schmautz et al., 2021). Bacteriophages can influence bacterial population dynamics and microbial community structure and have recently attracted attention as potential biological control agents for bacterial diseases in hydroponic production systems (Fortuna et al., 2023). Plant-associated viral sequences may also be introduced into hydroponic drainage systems through infected plant tissues, root release, or contaminated irrigation and drainage water (Mehle et al., 2023). However, active viral communities in hydroponic drainage systems remain poorly characterized, particularly in strawberry cultivation environments. Furthermore, interactions among active bacterial, fungal, and viral communities in hydroponic draining effluent remain poorly understood.

In this study, metatranscriptomic sequencing was performed to characterize the active microbial assemblages present in strawberry hydroponic drainage collected from a recirculating greenhouse cultivation system. Particular emphasis was placed on describing the taxonomic composition and structure of active bacterial and fungal communities, while viral-associated transcripts were evaluated as complementary components of the drainage ecosystem. We analyzed the taxonomic composition of bacterial, fungal, and viral-associated transcripts to characterize the active microbial community inhabiting the hydroponic drainage environment. Rather than attempting to define general characteristics of strawberry hydroponic microbiomes, this exploratory study provides a metatranscriptomic description of active microbial communities detected in drainage effluent from a commercial strawberry production system. Accordingly, the findings should be interpreted as site-specific observations rather than as representative of microbial community patterns across commercial strawberry hydroponic systems in general. The findings contribute site-specific information on active microbial assemblages inhabiting hydroponic drainage environments and provide a reference dataset for future comparative investigations of recirculating cultivation systems. This baseline dataset may facilitate future comparative studies involving multiple hydroponic production systems under different cultivation conditions.

## 2 Materials and methods

### 2.1 Sample collection

Hydroponic drainage effluent samples were collected from a commercial strawberry (*Fragaria × ananassa* Duch.) greenhouse operated under a recirculating hydroponic cultivation system located in Chuncheon-si, Gangwon-do, Republic of Korea. Drainage effluent discharged from strawberry cultivation beds was used as the sampling source for microbial community analysis.

Sampling was conducted during the spring (April 2023) and winter (December 2024) growing seasons. For each sampling event, 2 L of drainage effluent was collected directly from the drainage line into sterile containers and transported to the laboratory under chilled conditions.

To remove large plant debris and coarse suspended particles, the collected drainage effluent was first passed through sterile cheesecloth. To collect microbial biomass, each sample was vacuum-filtered through a sterile 0.22 μm polyethersulfone (PES) membrane filter (Merck Millipore, USA). The membranes were immediately transferred to sterile tubes and stored at −80 °C until RNA extraction.

### 2.2 RNA extraction, library preparation, and sequencing

Total RNA was extracted from PES membrane filters using the RNeasy^®^ PowerWater^®^ Kit (Qiagen, Hilden, Germany) according to the manufacturer’s instructions. Residual genomic DNA was removed by DNase I treatment (New England Biolabs, Ipswich, MA, USA) during the extraction procedure.

The quality and integrity of the extracted RNA were assessed using an Agilent 2100 BioAnalyzer (Agilent Technologies, Santa Clara, CA, USA). RNA samples meeting the quality requirements for sequencing were used for library preparation.

Prior to library construction, ribosomal RNA (rRNA) was depleted from the total RNA using the Illumina Ribo-Zero^™^ rRNA Removal Kit (Illumina, San Diego, CA, USA). Sequencing libraries were prepared by a commercial sequencing service provider (Invirustech, Gwangju, Republic of Korea). High-throughput sequencing was performed on an Illumina NovaSeq 6000 platform (Illumina, San Diego, CA, USA) using paired-end sequencing (2 × 151 bp). Raw sequence data were generated in FASTQ format and used for downstream bioinformatic analyses.

### 2.3 Bioinformatic analysis

Raw sequencing reads were assessed for quality using FastQC (v0.11.9). Adapter sequences and low-quality bases were removed using Trimmomatic (v0.39) with the parameters SLIDINGWINDOW:4:20 and MINLEN:50. Quality-filtered reads were subsequently used for downstream analyses.

*De novo* assembly was performed separately for each sample using MEGAHIT (v1.2.9) with default parameters. No host genome-based filtering was performed prior to assembly. The resulting contigs were used for taxonomic classification and microbial community characterization.

Taxonomic classification was conducted using Kraken2 (v2.1.2) with a custom database constructed from bacterial, archaeal, fungal, and viral genomes available in the NCBI RefSeq database.

To improve the accuracy of viral annotation, contigs assigned to viral taxa were further validated using BLASTx (v2.13.0) against the NCBI viral protein database and Minimap2 (v2.24). Viral contigs were retained when they met the following criteria: E-value < 1 × 10^−10^, query coverage > 50%, and contig length > 1,000 bp.

**Figure 1:**
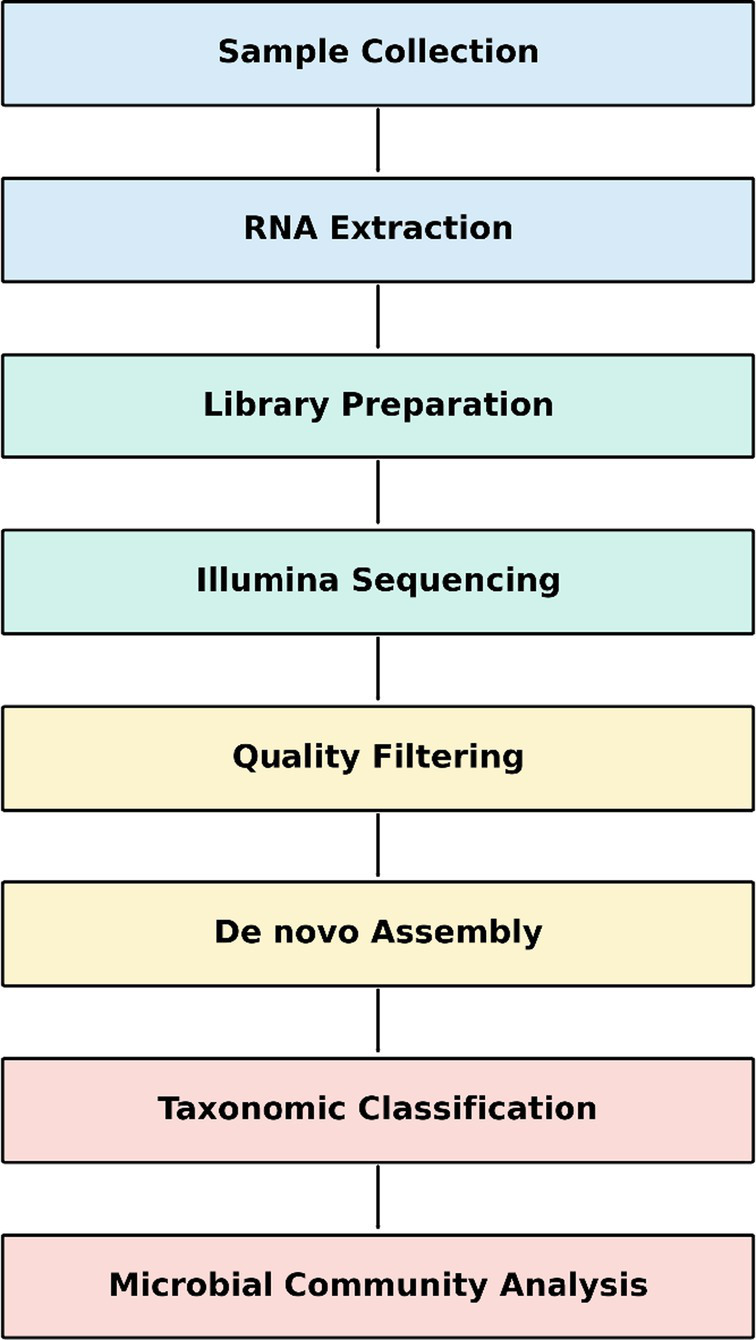
Workflow used for metatranscriptomic characterization of microbial communities in strawberry hydroponic drainage effluent. Hydroponic drainage effluent samples collected from strawberry cultivation beds in a commercial strawberry greenhouse were subjected to RNA extraction, library preparation, and Illumina sequencing. Quality-filtered reads were assembled *de novo* and taxonomically classified to characterize active microbial communities associated with the hydroponic drainage environment.

Taxonomic annotation results were processed and visualized using R (v4.2.2). Sankey diagrams, Krona plots, and stacked bar charts were generated to compare microbial and viral community composition between samples. An overview of the experimental design and bioinformatic workflow is presented in Figure 1.

## 3 Results

### 3.1 Sequencing overview

Metatranscriptomic sequencing generated a total of 65,150,876 and 53,159,798 paired-end reads from the spring and winter drainage effluent samples, respectively. The sequencing datasets yielded 9.77 Gb and 7.97 Gb of sequence data, with Q30 values exceeding 93% for both samples, indicating high sequencing quality. The GC contents of the spring and winter datasets were 59.90 and 60.97%, respectively.

Following quality filtering and *de novo* assembly, assembled contigs were used for downstream taxonomic classification and community analyses. The high sequencing depth and quality metrics obtained for both samples provided sufficient data for comprehensive characterization of active microbial communities associated with the hydroponic drainage environment. Detailed sequencing statistics and taxonomic classification summaries are presented in Table 1.

**Table 1:** Sequencing summary and taxonomic classification statistics of strawberry hydroponic drainage effluent samples.

Parameter	Spring	Winter
Raw reads	65,150,876	53,159,798
Data yield (Gb)	9.77	7.97
Q30 (%)	>93	>93
GC content (%)	59.90	60.97
Bacterial reads	7,614,899	12,458,940
Fungal reads	7,764	66,201
Viral reads	26,963	38,411
Protozoan reads	2,696	10,166
Unclassified reads (%)	87.65	47.58

*Values represent sequencing output and taxonomic classification results obtained from metatranscriptomic analysis of hydroponic drainage effluent samples collected during spring and winter cultivation periods.

*Read counts indicate the number of reads assigned to each microbial group. Percentages of unclassified reads were calculated relative to total sequencing reads.

### 3.2 Overall taxonomic composition of strawberry hydroponic drainage

Taxonomic classification revealed substantial differences in the composition of annotated transcripts between spring and winter drainage effluent samples.

In the spring sample, bacterial transcripts represented the majority of classified sequences, accounting for 7,614,899 reads, followed by viral (26,963 reads), fungal (7,764 reads), and protozoan (2,696 reads) sequences. However, a large proportion of reads (87.65%) remained unclassified.

In contrast, the winter sample contained 12,458,940 bacterial reads, 38,411 viral reads, 66,201 fungal reads, and 10,166 protozoan reads. The proportion of unclassified reads was markedly lower (47.58%) than that observed in the spring sample. Consequently, classified microbial transcripts accounted for a substantially larger fraction of the winter metatranscriptome than of the spring metatranscriptome.

**Figure 2:**
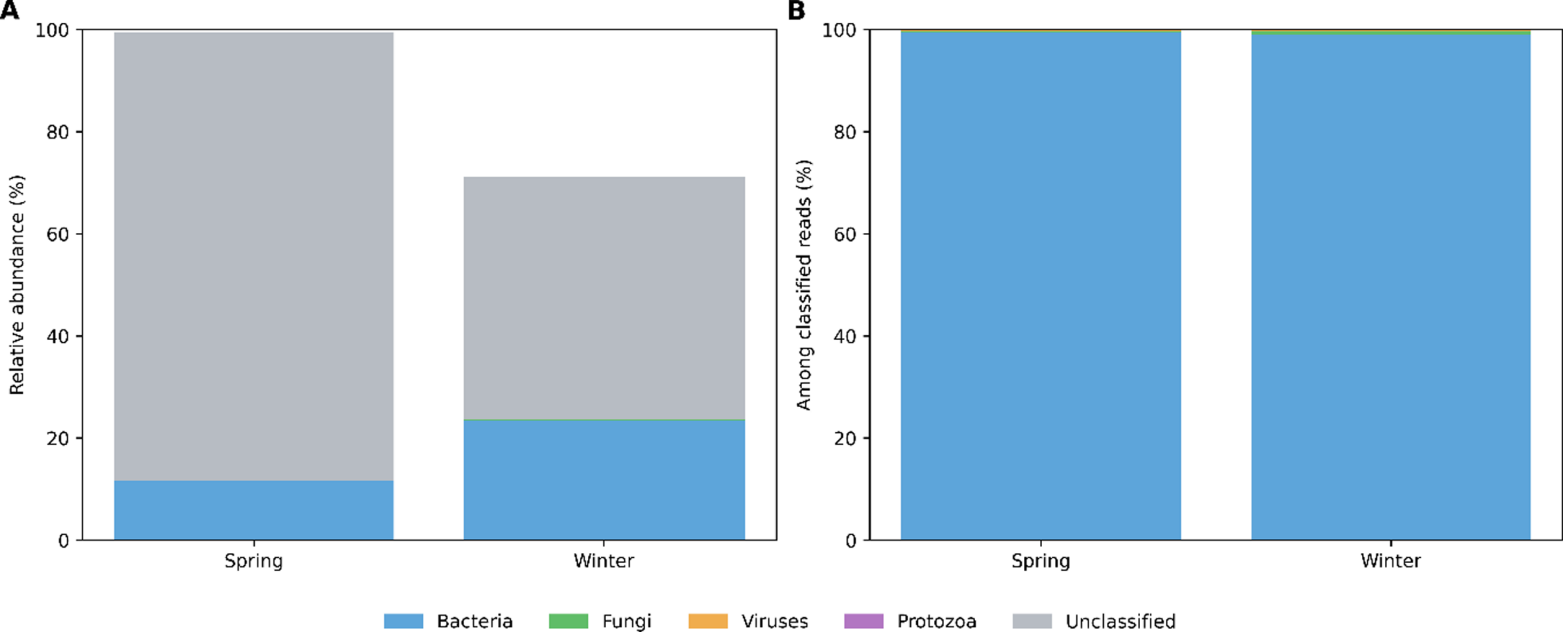
Overall taxonomic composition of metatranscriptomic reads detected in Strawberry hydroponic drainage effluent samples. **(A)** Relative abundance of bacterial, fungal, viral, protozoan, and unclassified reads based on total sequencing reads. **(B)** Relative abundance of bacterial, fungal, viral, and protozoan reads among classified reads only. Relative abundances are presented as percentages of sequencing reads detected in the spring and winter drainage samples.

The winter drainage sample exhibited a substantially higher proportion of classified microbial transcripts than the spring sample, indicating distinct taxonomic profiles between the two analyzed drainage effluent samples (Figure 2).

### 3.3 Bacterial community composition and dominant taxa

Bacterial communities differed considerably between spring and winter drainage samples.

The spring sample exhibited a relatively diverse bacterial community composed of multiple taxa with comparable abundances. The most abundant species included *Ferrovibrio terrae*, *Novosphingobium ginsenosidimutans*, *Opitutus* sp. GAS368, *Stella vacuolata*, and *Bosea* sp. ANAM02. No single bacterial taxon showed marked dominance in the spring sample, indicating a comparatively even community structure.

In contrast, the winter sample was characterized by strong dominance of *Serratia*-associated taxa. The most abundant bacterial species were *S. marcescens* (1,065,022 reads) and *S. proteamaculans* (599,122 reads), followed by *Rhodoferax* sp., *Raoultella* sp., and *Pantoea* sp. Together, the two *Serratia* species accounted for a substantial proportion of classified bacterial transcripts in the winter sample, indicating a strongly skewed community composition.

**Figure 3:**
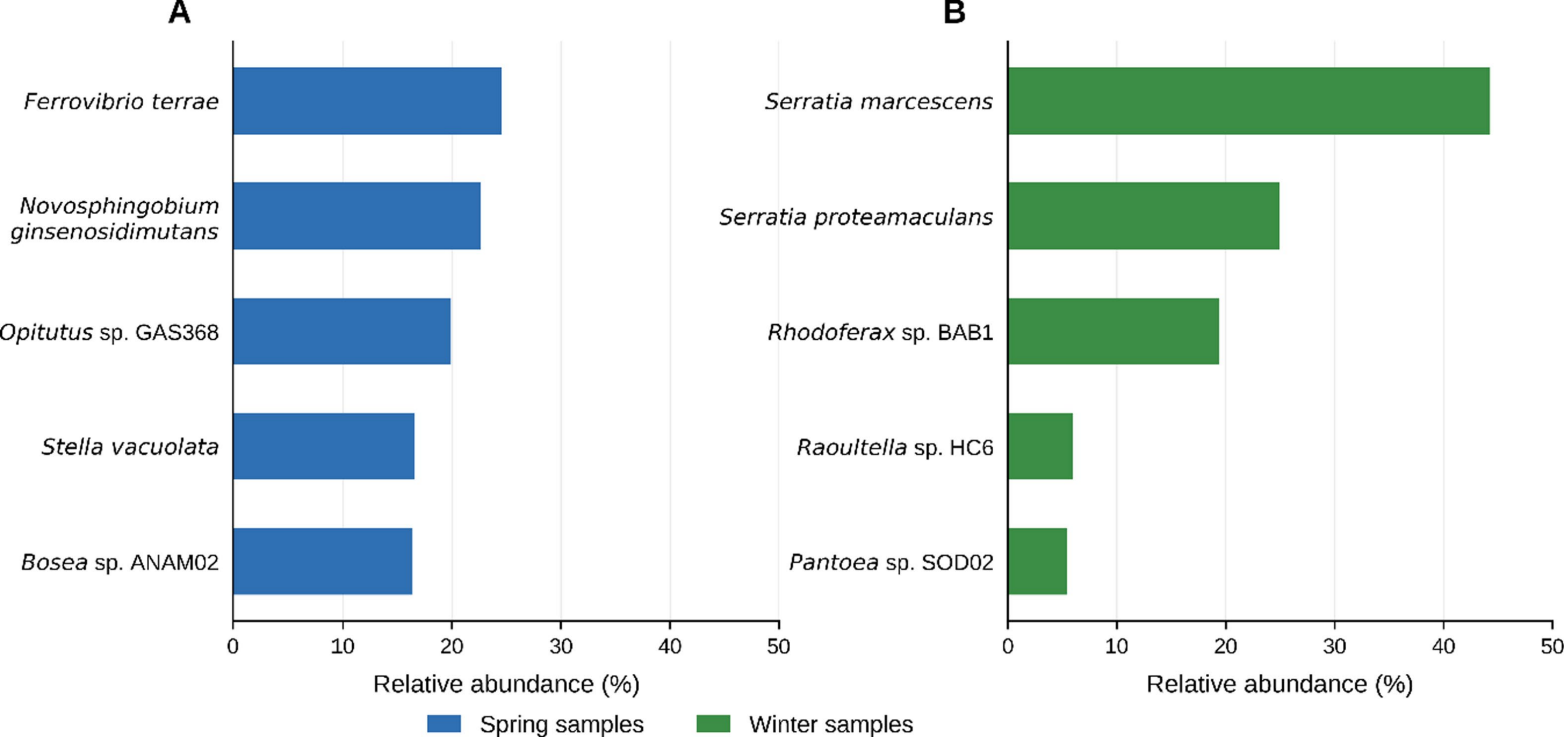
Relative abundance of dominant bacterial taxa detected in strawberry hydroponic drainage effluent samples. **(A)** Bacterial community composition of the spring drainage sample (blue bars). **(B)** Bacterial community composition of the winter drainage sample (green bars). The five most abundant bacterial taxa identified in each sample are shown. Relative abundances were calculated as percentages of total bacterial reads assigned to bacterial taxa within each sample.

Compared with the spring sample, the winter drainage effluent exhibited a less evenly distributed bacterial profile, with a substantial proportion of classified bacterial transcripts assigned to *Serratia* species. These results indicate that the bacterial communities detected in the two drainage samples were characterized by markedly different taxonomic structures (Figure 3).

### 3.4 Fungal community composition

Fungal community composition also differed between the two drainage samples. The spring sample was dominated by transcripts assigned to uncultured fungi, *Amorphotheca resinae*, *Rhizophagus irregularis*, *Epichloë typhina*, and *Pseudozyma flocculosa*. Several plant-associated fungal taxa were also detected at low abundance. The fungal community in the spring sample consisted of multiple taxa with no obvious dominance by a single fungal species.

In the winter sample, dominant fungal taxa included uncultured fungi, *Komagataella phaffii*, *Meira miltonrushii*, *Yarrowia lipolytica*, and *Plectosphaerella* species. Compared with the spring sample, the winter sample exhibited a greater abundance of yeast-associated fungal taxa. Several of the dominant winter taxa, including *K. phaffii* and *Y. lipolytica*, are commonly associated with yeast-like lifestyles, suggesting a fungal community composition distinct from that observed in the spring sample.

Transcripts associated with several plant-pathogenic fungi, including *Verticillium dahliae*, were also detected, although at relatively low abundance.

**Figure 4:**
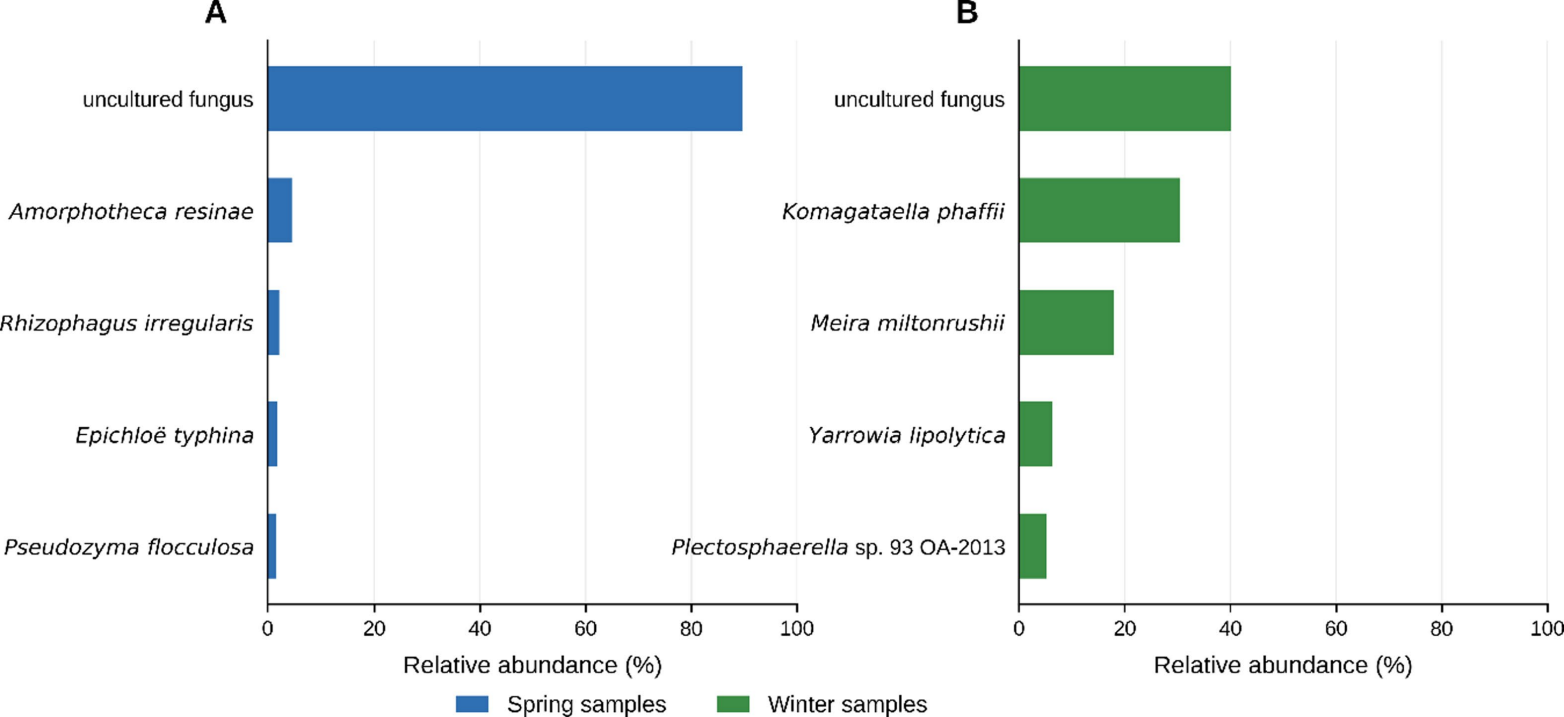
Relative abundance of dominant fungal taxa detected in strawberry hydroponic drainage effluent samples. **(A)** Fungal community composition of the spring drainage sample (blue bars). **(B)** Fungal community composition of the winter drainage sample (green bars). The five most abundant fungal taxa identified in each sample are shown. Relative abundances were calculated as percentages of total fungal reads assigned to fungal taxa within each sample.

The fungal communities detected in the two drainage samples exhibited distinct taxonomic compositions, with the winter sample showing a greater representation of yeast-associated fungi (Figure 4).

### 3.5 Viral-associated transcripts detected in drainage samples

Viral-associated transcripts were detected in both spring and winter drainage effluent samples.

The spring sample was dominated by bacteriophage-associated sequences, including members of the families *Leviviridae*, *Siphoviridae*, and Caudovirales-related phages. Additional viral taxa, including *Sphingomonas* phages and Andhravirus-related sequences, were also identified. Most viral sequences detected in the spring sample were associated with bacteriophages, indicating that bacteriophage-related transcripts represented a major component of the detected viral community.

In the winter sample, viral transcripts assigned to *Microviridae*, *Siphoviridae*, and uncultured Caudovirales phages were detected. Plant-associated viral sequences, including *Striga asiatica* dicistro-like virus 2 and Arabidopsis halleri dicistro-like virus 1, were also identified. As in the spring sample, bacteriophage-associated sequences represented a substantial proportion of the detected viral transcripts.

**Figure 5:**
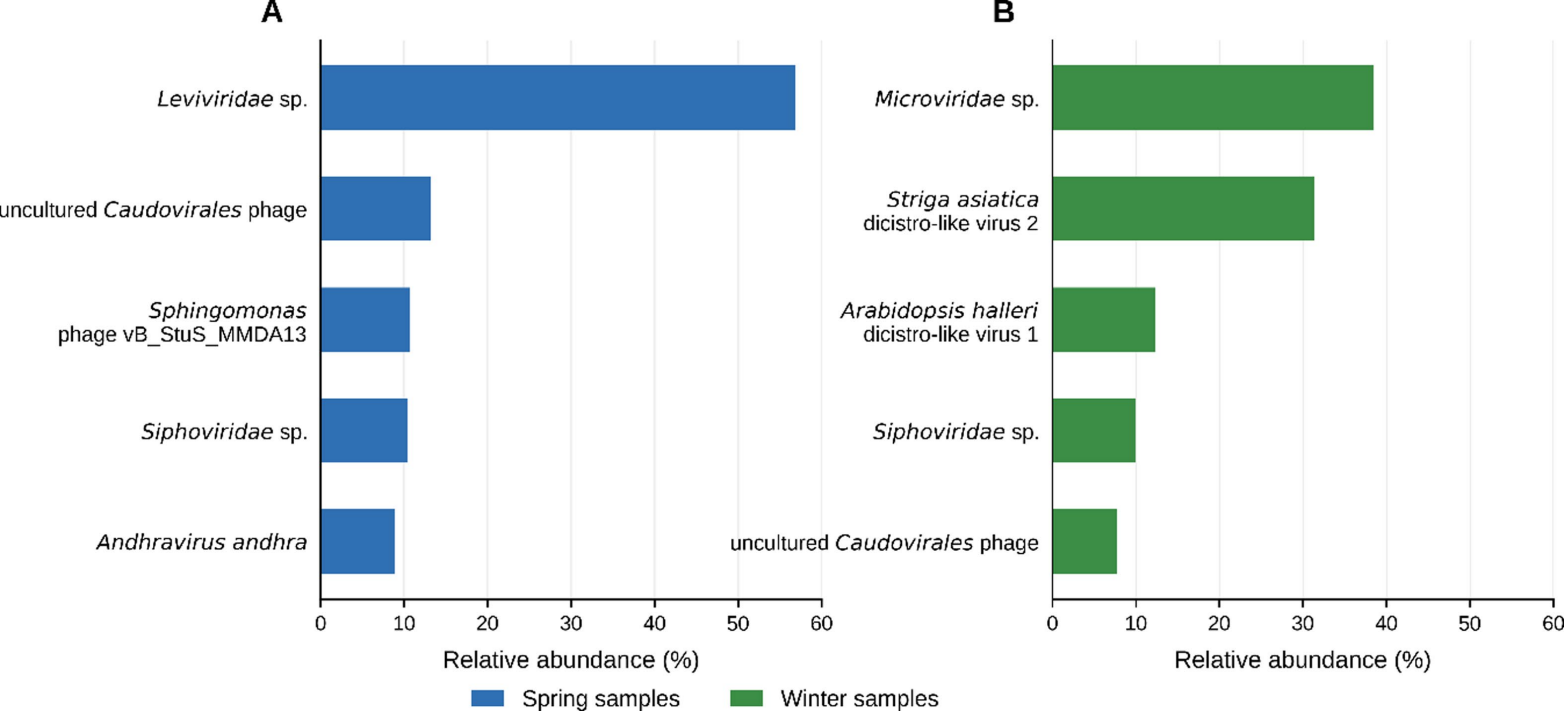
Relative abundance of dominant viral-associated taxa detected in strawberry hydroponic drainage effluent samples. **(A)** Viral-associated community composition of the spring drainage sample (blue bars). **(B)** Viral-associated community composition of the winter drainage sample (green bars). The five most abundant viral-associated taxa identified in each sample are shown. Relative abundances were calculated as percentages of total viral-associated reads assigned to viral taxa within each sample.

A total of 961 high-confidence viral contigs were recovered from the spring sample, whereas 306 high-confidence viral contigs were identified in the winter sample. Although a greater number of viral reads were detected in the winter sample, the spring sample yielded a larger number of assembled viral contigs. These findings indicate differences in the composition and assembly characteristics of viral-associated transcripts detected in the two drainage effluent samples (Figure 5).

The dominant bacterial, fungal, and viral taxa identified in the spring and winter drainage samples are summarized in Table 2.

**Table 2:** Dominant bacterial, fungal, and viral taxa detected in strawberry hydroponic drainage effluent samples.

Group	Rank	Spring	Winter
Bacteria	1	*Ferrovibrio terrae*	*Serratia marcescens*
	2	*Novosphingobium ginsenosidimutans*	*Serratia proteamaculans*
	3	*Opitutus* sp. GAS368	*Rhodoferax* sp. BAB1
	4	*Stella vacuolata*	*Raoultella* sp. HC6
	5	*Bosea* sp. ANAM02	*Pantoea* sp. SOD02
Fungi	1	uncultured fungus	uncultured fungus
	2	*Amorphotheca resinae*	*Komagataella phaffii*
	3	*Rhizophagus irregularis*	*Meira miltonrushii*
	4	*Epichloë typhina*	*Yarrowia lipolytica*
	5	*Pseudozyma flocculosa*	*Plectosphaerella* sp.
Viruses	1	*Leviviridae*	*Microviridae*
	2	uncultured Caudovirales phage	*Striga asiatica* dicistro-like virus 2
	3	Sphingomonas phage	Arabidopsis halleri dicistro-like virus 1
	4	*Siphoviridae*	*Siphoviridae*
	5	Andhravirus andhra	uncultured Caudovirales phage

*Only the five most abundant taxa identified in each sample are shown. Taxonomic assignments were based on Kraken2 classification of assembled contigs. Viral-associated sequences were further validated using BLASTx and Minimap2.

## 4 Discussion

The present study employed metatranscriptomic sequencing to characterize active microbial communities inhabiting hydroponic drainage effluent collected from a commercial strawberry production system. Active bacterial, fungal, and viral-associated transcripts were detected in both drainage samples, revealing distinct taxonomic profiles between the spring and winter sampling periods. Because only one composite sample was analyzed for each cultivation period, the observed differences should be interpreted as site-specific observations rather than statistically validated seasonal patterns. Nevertheless, the data provide an initial description of active microbial communities present in strawberry hydroponic drainage under commercial cultivation conditions.

One of the most notable findings was the marked difference in bacterial community composition between the two drainage samples. The spring sample exhibited a relatively even bacterial community structure, with several taxa including *F. terrae*, *N. ginsenosidimutans*, *Opitutus* sp. GAS368, and *Bosea* sp. contributing to the overall community profile. In contrast, the winter sample was strongly dominated by *S. marcescens* and *S. proteamaculans*. Members of the genus *Serratia* are commonly detected in soil, water, rhizosphere, and plant-associated environments and have been reported to exhibit diverse ecological functions, including nutrient cycling, biofilm formation, and interactions with plants and other microorganisms (Petersen and Tisa, 2013). The dominance of *Serratia*-associated transcripts observed in the winter sample indicates that these bacterial populations represented a major component of the active microbial community detected at the time of sampling. The observed differences between the spring and winter samples may suggest temporal variation in bacterial community. However, because only a single sample was analyzed for each cultivation period, the present data do not allow statistical evaluation of seasonal variation. Further studies involving repeated sampling across multiple spring and winter growing seasons will be necessary to clarify the environmental factors associated with these observations. A substantial proportion of sequencing reads remained unclassified, particularly in the spring sample, where more than 87% of reads could not be assigned to known taxa. Similar observations have been reported in environmental metatranscriptomic studies, where a large fraction of transcripts often originate from poorly characterized microorganisms or genomic regions that are not represented in current reference databases (Naumova et al., 2021; Kim et al., 2022). The high proportion of unclassified transcripts observed in the present study highlights the limited availability of reference sequences for microorganisms inhabiting hydroponic drainage environments and suggests that considerable unexplored microbial diversity may be present in these systems (Murali et al., 2018).

Fungal community composition also differed between the two drainage samples. The spring sample contained transcripts associated with diverse fungal taxa, including *A. resinae*, *R. irregularis*, and *P. flocculosa*, whereas the winter sample was characterized by a greater abundance of yeast-associated fungi such as *K. phaffii* and *Y. lipolytica* (Kutty and Philip, 2008). Yeast-associated fungi are frequently detected in aqueous and nutrient-rich environments where dissolved organic compounds are readily available. The increased representation of these taxa in the winter sample further highlights the distinct fungal community composition observed between the two analyzed drainage samples. A similar difference was also observed in the fungal community, with yeast-associated taxa being more abundant in the winter sample than in the spring sample. However, because only a single sample was analyzed for each cultivation period, these observations should not be interpreted as statistically validated seasonal patterns.

Several transcripts associated with plant-pathogenic fungi, including *V. dahliae*, were also detected, although at relatively low abundance. The detection of pathogen-associated transcripts does not necessarily indicate active disease development within the cultivation system, but it demonstrates the ability of metatranscriptomic analysis to detect a broad spectrum of fungal taxa present in hydroponic drainage environments (Vestrum et al., 2018). Such information may be useful for future studies investigating microbial monitoring strategies in recirculating hydroponic systems.

Viral-associated transcripts were detected in both drainage samples, with bacteriophage-related sequences representing a major component of the identified viral communities. Members of the families *Leviviridae*, *Siphoviridae*, *Microviridae*, and Caudovirales-related phages were identified across the two samples. Bacteriophages are common components of microbial ecosystems and were consistently detected in both drainage samples (Brown et al., 2022). In addition to bacteriophage-associated sequences, a small number of plant-associated viral transcripts were also detected. Because the primary objective of this study was to characterize active microbial communities rather than to comprehensively investigate viral diversity, the virome results should be considered complementary observations that provide additional insight into the complexity of hydroponic drainage ecosystems.

RNA-based metatranscriptomics allows the characterization of metabolically active microbial populations that may not be detected through culture-dependent approaches (Bashiardes et al., 2016; Shi et al., 2009). The present dataset therefore provides a valuable baseline resource for future investigations of microbial ecology and microbial monitoring in recirculating hydroponic cultivation systems.

The present study has several limitations. First, samples were obtained from a single commercial strawberry production site, limiting the generalizability of the findings. Second, only one composite drainage sample was analyzed for each cultivation period, precluding statistical comparisons and preventing robust evaluation of temporal or seasonal effects. Consequently, the differences observed between the spring and winter samples should not be interpreted as representative characteristics of strawberry hydroponic systems in general. Future studies incorporating multiple production sites, repeated sampling across multiple spring and winter growing seasons, and long-term monitoring will be necessary to determine whether the microbial patterns observed in this study are consistently associated with hydroponic strawberry cultivation. In addition, integrating metatranscriptomic analyses of plant tissues with drainage effluent samples would provide a more comprehensive understanding of microbial ecology and the relationships between plant-associated and drainage-associated microbial communities in recirculating hydroponic systems.

Despite these limitations, the present study provides an initial metatranscriptomic characterization of active microbial communities inhabiting drainage effluent from a commercial strawberry hydroponic system. The findings contribute site-specific baseline information regarding active bacterial, fungal, and viral-associated populations and provide a useful reference dataset for future investigations of microbial ecology in recirculating hydroponic cultivation environments.

## 5 Conclusion

This study provides an initial metatranscriptomic characterization of active microbial communities detected in drainage effluent from a commercial strawberry hydroponic system. Distinct bacterial, fungal, and viral-associated transcript profiles were observed between the two analyzed drainage samples, with *Serratia*-associated taxa dominating the winter sample and bacteriophage-related sequences representing a major component of the detected virome. Although the findings are limited to a single production site and should be interpreted as exploratory observations, the dataset provides useful baseline information for future investigations of microbial ecology and microbial monitoring in recirculating hydroponic cultivation systems.

## Data Availability

The datasets presented in this study can be found in the NCBI Sequence Read Archive (SRA) under BioProject accession number PRJNA1476331. The data are available at: https://www.ncbi.nlm.nih.gov/bioproject/PRJNA1476331.
